# A Targeted Sequencing Assay for Serotyping *Escherichia coli* Using AgriSeq Technology

**DOI:** 10.3389/fmicb.2020.627997

**Published:** 2021-01-15

**Authors:** Jacob R. Elder, Pina M. Fratamico, Yanhong Liu, David S. Needleman, Lori Bagi, Robert Tebbs, Adam Allred, Prasad Siddavatam, Haktan Suren, Krishna Reddy Gujjula, Chitrita DebRoy, Edward G. Dudley, Xianghe Yan

**Affiliations:** ^1^U. S. Department of Agriculture, Eastern Regional Research Center, Agricultural Research Service, Wyndmoor, PA, United States; ^2^Thermo Fisher Scientific, Genetic Sciences Division, Austin, TX, United States; ^3^Clear Labs, Menlo Park, CA, United States; ^4^E. coli Reference Center, The Pennsylvania State University, University Park, PA, United States

**Keywords:** targeted sequencing, molecular serotyping, *E coli*, AgriSeq technology, O-antigen

## Abstract

The gold standard method for serotyping *Escherichia coli* has relied on antisera-based typing of the O- and H-antigens, which is labor intensive and often unreliable. In the post-genomic era, sequence-based assays are potentially faster to provide results, could combine O-serogrouping and H-typing in a single test, and could simultaneously screen for the presence of other genetic markers of interest such as virulence factors. Whole genome sequencing is one approach; however, this method has limited multiplexing capabilities, and only a small fraction of the sequence is informative for subtyping or identifying virulence potential. A targeted, sequence-based assay and accompanying software for data analysis would be a great improvement over the currently available methods for serotyping. The purpose of this study was to develop a high-throughput, molecular method for serotyping *E. coli* by sequencing the genes that are required for production of O- and H-antigens, as well as to develop software for data analysis and serotype identification. To expand the utility of the assay, targets for the virulence factors, Shiga toxins (*stx*_1_, and *stx*_2_) and intimin (*eae*) were included. To validate the assay, genomic DNA was extracted from O-serogroup and H-type standard strains and from Shiga toxin-producing *E. coli*, the targeted regions were amplified, and then sequencing libraries were prepared from the amplified products followed by sequencing of the libraries on the Ion S5™ sequencer. The resulting sequence files were analyzed *via* the SeroType Caller™ software for identification of O-serogroup, H-type, and presence of *stx*_1_*, stx*_2_, and *eae*. We successfully identified 169 O-serogroups and 41 H-types. The assay also routinely detected the presence of *stx*_1a,c,d_ (3 of 3 strains), *stx*_2c−e,g_ (8 of 8 strains), *stx*_2f_ (1 strain), and *eae* (6 of 6 strains). Taken together, the high-throughput, sequence-based method presented here is a reliable alternative to antisera-based serotyping methods for *E. coli*.

## Introduction

The outer cell membrane of Gram-negative bacteria, including *E. coli*, contains the O-polysaccharide, also known as the O-antigen, which is a component of the lipopolysaccharide (LPS). The unique combination and arrangement of the repeating units of the sugar residues in the O-antigen portion of the LPS molecule are highly immunogenic and have been used to classify *E. coli* into serologically defined O-serogroups. Identification of O-serogroups is routinely performed in investigations of *E. coli* outbreaks as certain serogroups are associated with specific *E. coli* pathovars (e.g., serogroup O157 and enterohemorrhagic *E. coli*). Kauffmann (1947) first described the antigenic classification of *E. coli* based on the O-, K-, and H-antigens, and traditional serotyping by agglutination of antisera raised against boiled preparations of *E. coli* cultures has been the “gold standard” method since its development in the 1940's (Kauffmann, 1947; Orskov et al., 1977; Ørskov and Ørskov, 1984). A major drawback of this methodology is its unreliability. In our experience, up to a quarter of *E. coli* strains are considered “un-typeable” via this method due to the cross-reactivity of different serogroups, strains considered “rough” (i.e., do not express the O-antigen), and sometimes ambiguous reactivity of the antisera producing weak positive results. Identification of O-serogroup or H-type via the underlying genetic factors would circumvent misidentification associated with variability in display of the antigens on the cell surface.

In *E. coli*, the genes required for synthesis of the O-antigens are most often found in a cluster known as the O-antigen gene cluster (O-AGC) and located on the chromosome between the *gnd* and *galF* genes (Samuel and Reeves, 2003; DebRoy et al., 2016). There are two well recognized pathways for synthesis and extracellular display of *E. coli* O-antigens; the Wzx/Wzy pathway (Samuel and Reeves, 2003) and the Wzm/Wzt ABC transporter pathway (Greenfield and Whitfield, 2012). Of the 181 formally recognized O-serogroups, 93% produce O-antigens via the Wzx/Wzy pathway. This process has been most recently reviewed by Islam and Lam (2014) and begins by the Wzx flippase transferring the O-unit with the initial sugar attached from the cytoplasmic to periplasmic side of the cellular membrane. The Wzy polymerase then elongates the polysaccharide chain, and the Wzz enzyme regulates chain length and terminates polymerization. Finally, the WaaL ligase adds the O-antigen to the lipid A core to form a complete LPS molecule, which is exported to the surface. In the Wzm/Wzt pathway, the O-antigen is polymerized on the cytoplasmic face of the inner membrane before it is transferred across the membrane to the periplasmic space via a classical ABC transport system. The Wzm protein acts as a channel, and Wzt hydrolyzes ATP to drive transportation of the O-antigen across the cytoplasmic membrane (Greenfield and Whitfield, 2012). Due to the diversifying pressure imposed on the O-antigen by the host and other environmental factors, there is a high degree of heterogeneity in the sequences of the *wzx, wzy, wzm*, and *wzt*, making them ideal targets for DNA-based serogrouping assays.

*E. coli* H-types have been delineated by serological responses to flagellin, the protein subunit of the flagellum structure that is expressed on the cell surface. Of the 53 formally recognized H-types, 43 express flagellin encoded by the *fliC* gene, and ten H-types express flagellin *via* the genes *flkA, fllA, flmA*, and *flnA*. The *E. coli* H-types that express flagellin *via* an alternate locus usually retain an intact *fliC* gene in addition to the alternate flagellin gene. These alternate flagellar loci can encode genes that repress expression of the *fliC* locus, with the result being two flagellin genes with only one actively expressed on the cell surface (Ratiner, 1998; Feng et al., 2008). In other H-types with an alternate flagellin gene, mutations in the *fliC* gene prevent its functional expression (Tominaga, 2004; Tominaga and Kutsukake, 2007). The flagellin genes are highly polymorphic, and within the *fliC* gene, there is a central hypervariable region that is specific to each H-type. As with the O-AGC genes, the H-antigen subunit genes are under significant diversifying pressure, have a high degree of sequence heterogeneity, and are potential genetic markers for developing H-typing assays.

The serogroups originally identified as O31, O47, O67, O72, O94, and O122 have since been removed from the typing scheme as they were found to be repeats of previously identified O-serogroups, or isolates with these serogroups have since been identified as species other than *E. coli*. We had previously analyzed sequences representing all recognized *E. coli* O-AGC, and found that 29 O-serogroups were highly similar (98 to >99.9% identity) to one or more other O-serogroups (DebRoy et al., 2016) (Table 1) and are unlikely to be distinguished through sequencing-based assays. The serogroups OX6, OX9, OX10, OX13, OX19, OX21, OX25, OX28, OX38, and OX43 are considered provisional serogroups. We found that six of these provisional serogroups had a highly similar O-AGC sequence to previously identified O-serogroups (DebRoy et al., 2016). The serogroups O20 and O57 have O-AGC that are located outside of the *gnd*/*galF* locus and remain uncharacterized. Also, serogroup O14 is considered “rough” and does not produce an O-antigen. This leaves a total of 145 O-serogroups and 3 provisional O-serogroups that have sequenced O-AGC's that can potentially be distinguished by their sequences, as well as 20 clusters of two or more O-serogroups with highly similar O-AGC sequences (DebRoy et al., 2016).

**Table 1:** O-groups sharing >98% similarity in O-AGC sequence.

**Cluster number**	**Serogroups sharing O-AGC sequences^a^**
1	O2, O50
2	O11, OX19
3	O13*, O129*, O135*
4	O17, O44, O77, O106
5	O19*, OX43*
6	O28ac*, O42*
7	O46, O134
8	O90, O127
9	O101, O162
10	O107*, O117*
11	O124, O164
12	O128*, OX38*
13	O163*, OX21*
14	O168*, OX6*
15	O184, OX9

a *Asterisks indicate O-groups that were suggested to belong to the same O-group based on O-AGC similarity and antisera cross reactivity (DebRoy et al., 2016)*.

DNA sequence-based methods for serotype identification have been an area of focus for some time. Iguchi et al. (2015) developed PCR primers targeting unique sequences in the O-antigen gene clusters (O-AGC) of 162 O-serogroups. This method reliably identified O-serogroups, but serogrouping a subject strain by this method requires up to 20 multiplex PCR reactions. Another group, focusing on STEC serogroups, has described a similar multiplex PCR that identifies up to 137 O-serogroups, but it requires 14 PCR reactions (Ludwig et al., 2020). Restriction fragment length polymorphism combined with PCR has been proposed for O-serogrouping (Coimbra et al., 2000), but it is a less precise method than determining the exact sequence of the O-AGC, and there could be variation in the patterns observed across different laboratories. Microarrays for identification of O-serogroups have been developed by us and other groups (Liu and Fratamico, 2006; Lacher et al., 2014; Patel et al., 2016); however, this technology is becoming outdated as massively parallel sequencing platforms are more readily available. A web-based tool, SerotypeFinder (Joensen et al., 2015) (https://cge.cbs.dtu.dk/services/SerotypeFinder/) matches the O-antigen processing genes, *wzx, wzy, wzm*, and *wzt*, of the O-AGC of a subject strain to sequenced strains for determination of O-serogroup and similarly compares the *fliC, flkA, fllA, flmA*, and *flnA* for the identification of H-type. This method requires whole genome sequencing to determine O-serogroup, which limits the number of strains that can be serotyped in a single run, significantly adding to the cost and labor involved in using the system to determine serogroup.

In addition to improved reliability and high-throughput sample processing, a molecular serotyping assay could be greatly enhanced by also including detection of important virulence factors. Screening for virulence genes is considered routine as part of outbreak investigations and is performed for strain characterization. Shiga toxin-producing *E. coli* (STEC) strains that cause bloody diarrhea and hemolytic uremic syndrome typically carry genes encoding the Shiga toxins (*stx*_1_ and/or *stx*_2_), as well as the attachment protein, intimin (*eae*) (Boerlin et al., 1999; Jenkins et al., 2003; Werber et al., 2003; Ethelberg et al., 2004; Brooks et al., 2005; Gould et al., 2009; Naseer et al., 2017). With traditional or PCR-based serotyping, screening for virulence factors is done in separate assays. Also, this information would be found if a strain's genome was sequenced, but as mentioned above, this method is not high-throughput. The objective of this study was to develop a method that only requires a single amplification step followed by PCR product sequencing for the identification of most known *E. coli* O-groups and H-types and to screen for important virulence factors, while requiring minimal bioinformatic expertise for data analysis. Additionally, we chose the AgriSeq platform that allows for the multiplexing of samples in a single run to reduce labor and cost per sample. With this system, we were able to amplify, sequence, and reliably identify 169 O-serogroups, 41 H-types, as well as 3 *stx*_1_ variants, 5 *stx*_2_ variants, and *eae* from 6 strains.

## Materials and Methods

### Design of Primer Sequences

The process for designing primers targeting O-group-specific genes was performed as follows. First, O-AGC sequences were clustered using Cd-hit (Li and Godzik, 2006) in order to get a sense for the degree of similarity among the sequences. In the case where sequences were highly similar (>95% similarity), one representative sequence (the longest sequence) was chosen to represent the cluster. The representative sequences were cut into short substrings in order to identify k-mers (k = 12) that were unique to each O-AGC. Processing of these k-mers was done using a multi-threaded hash table (Marcais and Kingsford, 2011). k-mers that were shared between O-AGCs were filtered out such that only k-mers that were unique to a particular O-AGC were used in subsequent steps. These unique k-mers were mapped back to their derivative O-AGCs using the Torrent Mapping Alignment Program (https://github.com/iontorrent/TS/tree/master/Analysis/TMAP), and “signatures” representing these O-AGCs were identified as regions harboring a large concentration of these unique k-mers. Primers were designed against these signatures using scripts based on AmpliSeq Designer software (Ampliseq.com). In order to confirm uniqueness, primer pairs were aligned to (i) the original O-AGC sequences, as well as (ii) a set of *Enterobacteriaceae* genomes from GenBank. Primers were chosen for the final panel which satisfied two criteria: (i) that they were specific for a given O-AGC and (ii) that they did not yield any off-target alignments when aligned to the set of *Enterobacteriaceae* genomes. In the case where multiple primer pairs for a given O-AGC passed the filters described, primer pairs targeting the *wzx* and *wzy* genes were preferentially chosen to represent that O-AGC in the panel. For the O-groups O16, O149, O178, and OX28, primers were designed to genes other than *wzx* and *wzy* so as to meet the previously described criteria. The serogroups O52, O60, O92, O95, O97, and O99 produced an O-antigen *via* an alternate ABC transporter-dependent pathway, and the genes *wzm* and *wzt* were preferentially chosen. The serogroups O89 and O101 also produce an O-antigen *via* the ABC-transporter-dependent pathway; however, other genes were chosen to meet the criteria described above. Out of the 149 genes used for O-serogroup primer design, 88 were *wzx*, 49 were *wzy*, 3 were *wzm*, 3 were *wzt*, and 6 putative genes were targeted. The genes used to target each O-serogroup and accession numbers for each sequence are listed in Supplementary Table 1.

A similar process was followed for design of primers targeting H-type regions. A total of 42 primer sets were designed, most targeting the *fliC* flagellin subunit gene. The H-types H3, H47, H54, and H55 express alternate flagellin subunit genes while the *fliC* gene is silenced. The genes *flkA, flkA, flmA*, and *fllA*, respectively, were targeted for the H-types, as shown in Supplementary Table 2. Two primer sets were designed for the H28 *fliC* gene for the purpose of identifying all variants of this gene, as some strains have deletions in the central part of the gene, based on available sequence.

For the STEC virulence factors, our objective was to detect as many of the *stx* subtypes possible with as few primer pairs as possible, instead of differentiating the subtypes. To this aim, we also compiled sequences of the STEC virulence genes, *stx*_1_, *stx*_2_, and *eae* (Supplementary Table 3) and made multiple sequence alignments for the purpose of identifying conserved regions so that primers could be designed against these regions. A total of four primer sets were designed; one targeting all *stx*_1_ subtypes (*stx*_1a_, *stx*_1c_, and *stx*_1d_), 1 for *stx*_2a−e_ and *stx*_2g_, one for *stx*_2f_, and one for all recognized *eae* subtypes. Multiple sequence alignment determined the assay detects all *eae* subtypes listed in the NCBI database including alpha-1, alpha-2, alpha-8, beta-1, beta-2, beta-3, gamma-1, gamma-3, gamma-4, gamma-5, gamma-6, gamma-variant, delta, epsilon-1, epsilon-2, epsilon-3, epsilon-4, epsilon-6, epsilon-7, epsilon-8, zeta, zeta-2, zeta-3, eta, eta-2, theta, theta-2, theta-variant, iota-1, iota-1A, iota-1B, iota-1C, iota 2, kappa, lambda, mu, nu, xi, omicron, pi, rho, rho-2, rho-3, tau, and upsilon. *In situ* analysis showed the *stx*_1_ assay detects *stx*_1a_, *stx*_1c_, and *stx*_1d_. Two assays were designed to detect all *stx*_2_ targets. *In situ* analysis showed the *stx*_2_ assay 1 detects *stx*_2a_, *stx*_2b_, *stx*_2c_, *stx*_2d_, *stx*_2e_, *stx*_2g_, *stx*_2h_, *stx*_2i_, and *stx*_2k_; and the second *stx*_2_ assay detects *stx*_2f_. The subtypes *stx*_2h_, *stx*_2i_, and *stx*_2k_ have recently been identified (Bai et al., 2018; Martin et al., 2019; Yang et al., 2020), but little is known about their prevalence and distribution in STEC strains. Also, we designed a primer set targeting a conserved region in the *E. coli* and *Shigella* genomes between the genes *malE* and *malK*, of the maltose transport system, for the purpose of confirming that a tested strain was in fact *E. coli*. Lastly, we designed positive control primers targeting regions in the *Thermatoga maritima* genome. The primers were designed to specifically target the internal positive control DNA that could be spiked to the tested samples.

### Molecular Serotyping and Detection of Virulence Factors

O-serogroup and H-type standard strains representing 169 O-serogroups and 41 H-types were tested (Supplementary Tables 1, 2). We also selected 12 STEC strains that carried variants of *stx*_1_ (3 strains), *stx*_2_ (8 strains), and *eae* (6 strains) (Table 2). An exclusivity panel of strains representing *Citrobacter freundii, Klebsiella pneumoniae, Yersinia enterocolitica, Pseudomonas fluorescens, Shigella boydii, Shigella sonnei*, and *Shigella flexneri* was also compiled (Table 3). Overnight cultures were grown in tryptic soy broth at 37°C at 150 rpm. DNA was extracted with the DNeasy kit (Qiagen), and DNA concentration was measured with the Qubit (Invitrogen). DNA concentrations were normalized to 10 ng/μL and spiked with positive control DNA for a total of 30 ng of *E. coli* DNA and 0.1 ng of *T. maritima* strain MSB8 (ATCC) DNA per 10 μL reaction. Amplified libraries for targeted sequencing were generated with the AgriSeq^TM^ platform. AgriSeq is based on the AmpliSeq targeted sequencing technology. AgriSeq was developed for non-clinical applications, with a primary focus on agri-genomic applications. The AgriSeq HTS Kit reagents (catalog number A34143, Thermo Fisher Scientific, Inc., Waltham, MA) are classified as research use only, and not for use in diagnostic procedures. The AgriSeq workflow has been adjusted to allow for high-throughput sample processing. Normalized DNA was combined with the commercially available *E. coli* Genoserotyping GBS Panel (available for purchase by contacting agriseq.gbspanel@thermofisher.com, Thermo Fisher Scientific) and AgriSeq™ Amplification Mix (Thermo Fisher). For amplification of genomic targets, the following thermocycling program was used: 99°C for 2 min, and then 21 cycles of 99°C for 15 s and 60°C for 4 min. Amplicons were then digested with the Pre-ligation Enzyme (Thermo Fisher Scientific) at 50°C for 10 min, 55°C for 10 min, and 60°C for 20 min. IonCode™ Barcode Adapters 1-384 Kit (catalog number A29751, Thermo Fisher Scientific) were ligated to the digested products with Barcoding Enzyme (Thermo Fisher Scientific). Labeled amplicons were pooled, equalized, amplified, and purified *via* the AgriSeq™ HTS Library Kit (Thermo Fisher Scientific) before loading onto an Ion 540™ sequencing Chip Kit (catalog number A27765, Thermo Fisher Scientific) *via* the Ion 540™ Chef kit (catalog number A30011, Thermo Fisher Scientific) and Ion Chef™ (Thermo Fisher Scientific). Sequencing was then performed on the Ion S5 system (Thermo Fisher Scientific).

**Table 2:** AgriSeq results for virulence factor screening from STEC strains.

**STEC strain serotype**	**Previously identified virulence factors**	**AgriSeq call**				
		***E. coli* spp**.	***stx*_**1**_**	***stx*_**2**_**	***stx*_**2f**_**	***eae***
O15:H27	*stx*_1c_, *stx*_2d_	+	+	+	–	–
O121:H10	*stx*_2e_	+	–	+	–	–
O2:H25	*stx*_2g_	–	–	+	–	–
O145:NM	*stx*_2d_, *eae*	+	–	+	–	+
O157:H7	*stx*_2c_, *eae*	+	–	+	–	+
O26:H11	*stx*_1a_, *eae*	+	+	–	–	+
O121 (1)	*eae*	+	–	–	–	+
O121 (2)	*eae*	+	–	–	–	+
O138	*stx*_2e_	+	–	+	–	–
O41:H26	*stx*_1d_	+	+	–	–	–
O177:H25	*stx*_2c_, *stx*_2d_, *eae*	+	–	+	–	+
O63:H6	*stx*_2f_, *eae*	+	–	–	+	+

**Table 3:** AgriSeq results for exclusivity panel of non-*E. coli* strains.

**Species**	**O-group detected**	**H-type detected**	***E. coli* spp**.
*Citrobacter freundii*	None	None	–
*Klebsiella pneumoniae*	None	None	–
*Yersinia enterocolitica*	None	None	–
*Pseudomonas fluorescens*	None	None	–
*Shigella boydii*	O149	None	–
*Shigella sonnei*	None	None	+
*Shigella flexneri*	O13/O129/O135	None	+

### Data Analysis

**Figure 1:**
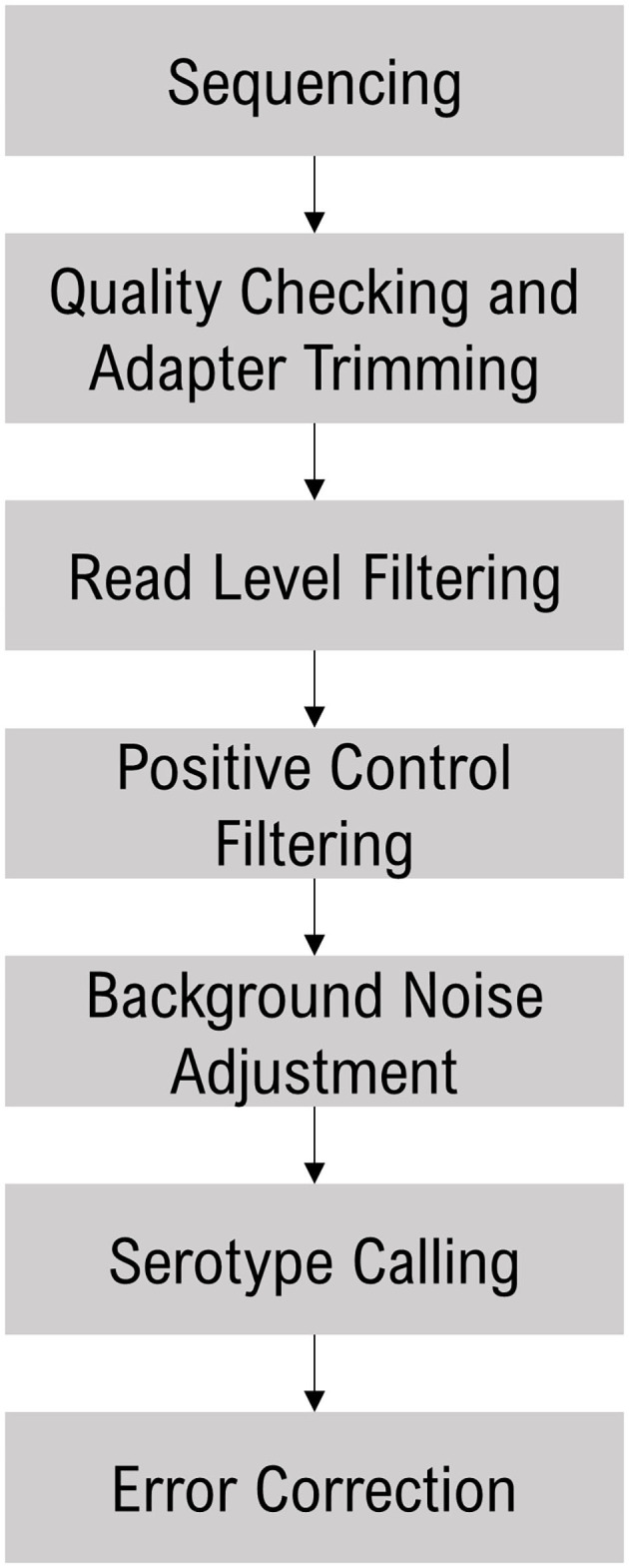
SeroTyper pipeline for calling *E. coli* serotypes and *stx* and *eae* genes.

The sequence data were analyzed with the Torrent Suite Software (TSS - https://github.com/iontorrent/TS), which was developed to handle the IonTorrent sequencing results. The sequencing reads were mapped to the target *E. coli* amplicon regions using the TMAP tool. The resulting BAM and BAI files were analyzed *via* the proprietary SeroTyper software pipeline (available by contacting agriseq.gbspanel@thermofisher.com). The mapped reads are filtered out based on three criteria; (i) read length <40 nucleotides, (ii) exceeding eight mismatches in alignment with the reference sequence, and (iii) read coverage of <70% of the amplicon length. The SeroTyper plugin uses several heuristic filters for calling serotypes, and the workflow is detailed in Figure 1. Briefly, the reads mapped to the TM positive controls are used to exclude the samples with a z-score threshold of −1.2. In order to adjust for the background noise, serotypes with a read threshold of <20 are excluded from the downstream analysis. Serotype calling is performed with a simple classification technique suitable for single dimensional data, the Jenks natural breaks algorithm (Jenks, 1967). This algorithm groups the samples by decreasing the variance within a group and increasing variance between the groups. This technique works well for identifying the mixed samples with multiple O- and H-types within a sample. Finally, error correction is performed to eliminate the potential false positive calls with a principal component analysis clustering algorithm. *E. coli* species and *stx* and *eae* genes are called with mapped read percentages at >1% of the total reads.

## Results and Discussion

### *E. coli* O-serogrouping

As described above, traditional O-serogrouping is a tedious and unreliable method of subtyping *E. coli*. Targeted sequencing of the highly variable O-AGC could present a rapid and reliable alternative to traditional O-serogrouping. Our goal in this study was to develop a targeted sequencing assay that was rapid, reliable, and user friendly to fill this role. We began by compiling the O-AGC sequences for 161 O-serogroups and 8 provisional OX-serogroups. We identified a total of 149 unique O-AGC regions among the 169 O- and OX-serogroups *via* the process described above, and then subsequently designed primers targeting these regions. The primers were then validated on reference strains representing each of the 169 O- and OX-serogroups. Overnight cultures of each strain were used for purification of genomic DNA three times for each reference strain to serve as template for amplification of the O-AGC target regions. The amplified target regions were sequenced, and the resulting sequences were analyzed with the proprietary SeroTyper software pipeline for O-serogroup calling. We successfully amplified the O-AGC targets and confirmed the O-serogroup of all 169 reference strains using three independent replicates per strain (Supplementary Table 1).

Although the O-AGC sequence is correlated with the structure and therefore the serology of the O-antigen, this relationship is not perfect as genes outside of the O-AGC can affect the O-antigen structure (DebRoy et al., 2016). Correlating O-AGC sequence with serology of the O-antigen is further complicated by the stochastic nature of the production of polyclonal antisera against O-antigens. While characterizing strains with nearly identical O-AGC sequences, different groups have found different patterns of antisera cross-reactivity (Joensen et al., 2015; DebRoy et al., 2016). In the current study, 33 serogroups could not be parsed to the level of individual serogroups and were indistinguishable serogroups based on sequence of the O-AGC. Out of the 33 serogroups, we had previously proposed that 15 serogroups should be collapsed into 7 serogroups based on highly similar O-AGC (>98% identity) and serological cross-reactivity (as shown in Table 1) based on O-AGC sequence and serological cross-reactivity (DebRoy et al., 2016). This leaves 18 serogroups comprising 8 O-AGC clusters that cannot be completely resolved *via* sequences in the O-AGC. It is debatable which differences in O-antigens reliably indicate strains that are different in a biologically meaningful way.

### *E. coli* H-typing Results

An advantage of the AgriSeq technology used in the present study is that it allows for the inclusion of up to tens of thousands of primer pairs, allowing us to combine O-serogrouping and H-typing in a single test. For the addition of H-types, we took a similar approach to the one used for O-serogroups and started by identifying target regions specific to each H-type. As described above, the flagellar subunit genes are heterogenous and represent good targets for this purpose. We compiled sequences of the genes encoding the flagellin gene for 41 H-types and selected target regions as described above. Most *E. coli* H-types are associated with expression of the flagellar subunit from the *fliC* gene (Wang et al., 2003). For 37 of the H-types included in this assay, the target regions were located in *fliC* and were successfully amplified and sequenced (Supplementary Table 2). The H-types, H3, H47, H54, and H55, have a *fliC* gene, but it is not expressed due to mechanisms described below. In these H-types, the expressed flagellar subunits are encoded by the alternate genes: *flkA* in H3 and H47, *flmA* in H54, and *fllA* in H55. The *E. coli* strains belonging to the serotypes H3 and H47, harbor a *flk*-genomic islet. This islet, in addition to the *flkA*, flagellar subunit gene, encodes *flkB*, a repressor of *fliC* (Feng et al., 2008). This results in both *flkA* and *fliC* being maintained in the genome but only *flkA* being expressed (Feng et al., 2008). How this affects the AgriSeq assay is that both the *flkA* and *fliC* genes are detected, resulting in two H-type calls for the H-types H3 and H47. In the case of H-type H55, the *fllA* gene is expressed while point mutations in the promoter regions of *fliC*_H38_ results in the repression of the *fliC*_H38_ gene (Tominaga and Kutsukake, 2007). The H-type H54, has flagellin encoded by the *flmA* gene and has an insertion element that disrupts and prevents functional expression from *fliC*_H21_ (Tominaga, 2004). As observed with H3 and H47, the *fliC* genes and the alternate flagellar subunit genes are detected by the AgriSeq assay.

### Virulence Factors and *E. coli* spp. Locus

In addition to O-group and H-type, the presence of virulence factors is valuable information in surveillance of *E. coli* outbreak strains. As mentioned above, we chose to focus on the virulence factors associated with STEC. We compiled sequences of *stx*_1_, *stx*_2_, and *eae*, (Supplementary Table 3) that encode the Shiga-toxin genes and intimin gene, respectively. Consensus sequences were used for designing primers that would detect all known *stx*_1_, *stx*_2_, and *eae* subtypes. A separate set of primers were required for amplification of *stx*_2f_ as this variant differs significantly from the other subtypes (Schmidt et al., 2000). We tested each subtype and successfully identified these genes in 3/3, 8/8, 1/1, and 6/6 of strains that carried *stx*_1_, *stx*_2c−e, g_, *stx*_2f_, and *eae*, respectively (Table 2).

Lastly, we designed a set of primers for an intergenic region between the *malF* and *malE* genes that is specific to the *E. coli* species. The purpose of these primers was to identify strains as *E. coli* and distinguish them from other *Enterobacteriaceae* species that share the same O-antigen genes. It has been shown that *Citrobacter, Klebsiella*, and *Salmonella*, among other genera in the family, can have highly similar O- and H-antigens to *E. coli* (Jansson et al., 1985; Samuel et al., 2004; Hu et al., 2010). This locus was detected in 99% of *E. coli* replicates tested. The OX38 and O2:H50 STEC strains were the only *E. coli* strains to not show amplification of the *E. coli* species target (Supplementary Table 1 and Table 2) and due to the limited sequence information for OX38 and the O2:H50 strain, it is unclear why these strains are not positive. Whole genome sequencing of OX38 strains and the O2:H50 strain may be required to characterize the region where these primers target. We included an exclusivity panel that showed that *Citrobacter freundii, Shigella boydii, Salmonella enterica* serovar Typhimurium*, Klebsiella pneumoniae, Yersinia enterocolitica*, and *Pseudomonas fluorescens* were not positive for this locus and could therefore be differentiated from *E. coli* (Table 3). The species *Shigella sonnei* and *Shigella flexneri* were positive for this locus (Table 3). This is not unexpected due to the close phylogenetic relationship between *Shigella* and *E. coli*. The *Shigella flexneri* isolate was also positive for the O13/O129/0135 O-AGC target. A high degree of similarity has been previously observed between the O-AGC of *Shigella flexneri* type 5a and the O-AGC of *E. coli* O-serogroups O13, O129, and O135 (Perepelov et al., 2010). The *Shigella boydii* strain used for this study was positive for O149. This is also not unexpected as the *Shigella boydii* O1 is known to have an identical O-AGC to *E. coli* O149 (Tao et al., 2005).

In this study we included the STEC virulence factors *stx* and *eae*, however extra-intestinal pathogenic *E. coli* (ExPEC) and other *E. coli* pathovars, with distinct sets of virulence factors, are well-recognized as important pathogens. Virulence factors from other *E. coli* pathovars, as well as additional STEC virulence factors, could be included in future versions of this assay. The AgriSeq technology on which this assay is based, has the potential capacity to include up to 24,000 primer pairs in a single panel, which allows this assay to adapt as additional serotypes or virulence factors are discovered. Also, this system has high multiplexing capacity, accommodating up to 768 samples in a single run with the IonCodeTM barcode adapters, although for this work, we typically combined 192 samples per run. Processing the same number of samples using whole genome sequencing, while providing more information, would require ten to one hundred-fold more sequencing runs and the cost per strain would be significantly higher. A main objective of our work was to develop a cost-effective, high-throughput, and accurate method for molecular serotyping of *E. coli*.

One limitation of the current study is that only O- and H-standard strains were used for validation of the method. It is possible that strain to strain sequence variations within the same O- or H-type could affect the amplification of O- and H-type primers. Whenever possible, we used multiple reference sequences to design primers and avoided areas where polymorphisms were found in a single O- or H-type. Additional work to validate the assay on field isolates and other strains is needed, and this work is ongoing in our laboratory.

We present here a rapid and reliable *E. coli* molecular serotyping assay that we have demonstrated can identify 169 of O-groups and 41 H-types. We believe the technology presented here can be successfully implemented to identify the remaining 28 O-groups and 12 H-types; work to include these is currently on-going. A relatively small number of *E. coli* serogroups have been associated with the majority of STEC outbreaks in humans, including O157, O26, O111, O103, O121, O145, and O45 (Valilis et al., 2018). Other serotypes have been detected at lower frequencies; however, it is possible, if not likely, that strains of STEC belonging to other serotypes are underappreciated due to biases in surveillance and testing (Valilis et al., 2018). Reliable and accessible serotyping platforms for *E. coli* are needed to better understand the human disease burden of these underappreciated serotypes. The assay presented here could fill such a role.

## Data Availability Statement

The original contributions presented in the study are included in the article/Supplementary Material, further inquiries can be directed to the corresponding author/s.

## Author Contributions

JE extracted DNA, prepared and sequenced libraries, and drafted the manuscript. PF conceived of the study, compiled sequence data, contributed to discussions of the results, and helped draft the manuscript. LB extracted DNA and helped prepare and sequence libraries. RT conceived of the study, helped in sequencing *E. coli* genomes and compiling sequence data, performed data analysis, contributed to discussions of the results, and helped draft the manuscript. YL contributed to discussions of the results and helped draft the manuscript. DN contributed to discussions of the results. PS, HS, and KG analyzed the sequencing data, designed the serotype calling algorithm, and designed the SeroTyper software. CD and ED provided genomic DNA and contributed to discussions of the results. XY helped to compile sequence data and design primers. All authors contributed to the article and approved the submitted version.

## Conflict of Interest

The authors declare that the research was conducted in the absence of any commercial or financial relationships that could be construed as a potential conflict of interest.
