# Supplementary material for: A Targeted Sequencing Assay for Serotyping Escherichia coli Using AgriSeq Technology
Source: Front Microbiol. 2021 Jan 15;11:627997. doi: 10.3389/fmicb.2020.627997 (PMC7844058; doi:10.3389/fmicb.2020.627997)
Supplement: Supplementary file 1 [file Data_Sheet_1.docx]

Supplementary Material

# Supplementary Tables

**Supplementary Table S1.** AgriSeq O-serogrouping results of O-group standard strains.

| Strain no. | Strain designation | Target gene | Accession no. | Serotype | AgriSeq call | *E. coli* spp. |
| --- | --- | --- | --- | --- | --- | --- |
| O001 | U5-41 | O1-wzx | GU299791 | O1:K1:H7 | O1 | + |
| O002 | U9-41 | O2-wzy | EU549863 | O2:K1:H4 | O2, O50 | + |
| O003 | U14-41 | O3-wzx | EU694097 | O3:K2ab:H2 | O3 | + |
| O004 | U4-41 | O4-wzx | AY568960 | O4:K3:H5 | O4 | + |
| O005 | U1-41 | O5-wzx | KP710588 | O5:K4:H4 | O5 | + |
| O006 | Bi7458-41 | O6-wzx | AJ426045 | O6:K2a:H1 | O6 | + |
| O008 | G3404-41 | O8-wzx | AB811598 | O8:K8:H4 | O8 | + |
| O010 | Bi8337-41 | O10-wzx | KJ755557 | O10:K5:H4 | O10 | + |
| O011 | Bi623-42 | O11-wzx | HQ388393 | O11:K10:H10 | OX19, O11 | + |
| O012 | Bi626-42 | O12-wzx | KJ755558 | O12:K5:H- | O12 | + |
| O013 | Su4321-41 | O13-wzy | EU296422 | O13:K11:H11 | O129, O13, O135 | + |
| O015 | F7902-41 | O15-wzx | AY647261 | O15:K14:H4 | O15 | + |
| O016 | F11119-41 | O16-rfbA, rfbC | HQ388392 | O16:K1:H- | O16 | + |
| O017 | K12a | O106-wzx | AB812084 | O17:K16:H18 | O106, O17, O44, O73, O77 | + |
| O018 | F10018-41 | O18-wzx | GU299793 | O18ab:K-:H14 | O18 | + |
| O018 | F10018-41 | O18-wzx | AB811603 | O18ab:K-:H14 | O18 | + |
| O019 | F8188-41 | O19-wzx | AB811604 | O19ab:K-:H7 | O19, OX43 | + |
| O020 | P7a | O20-wzx | KJ778793 | O20:K17:H- | O20 | + |
| O021 | E19a | O21-wzy | EU694098 | O21:K20:H- | O21 | + |
| O023 | E39a | O23-wzx | KJ755561 | O23:K18ab:H15 | O23 | + |
| O024 | E41a | O24-wzy | KJ755562 | O24:K+:H- | O24 | + |
| O027 | F9884-41 | O27-wzx | GU014555 | O27:K-:H- | O27 | + |
| O028ab | K1a | O28ab-wzy | KP710590 | O28ab:K-:H- | O28ab | + |
| O028ac | Kattwijk | O28ac-wzy | DQ462205 | O28ac:K?:H- | O28ac, O42 | + |
| O029 | Su4338-41 | O29-wzx | EU294173 | O29:K-:H10 | O29 | + |
| O030 | P2a | O30-wzy | KJ755563 | O30:K-:H- | O30 | + |
| O032 | P6a | O32-wzy | EU296410 | O32:K-:H19 | O32 | + |
| O033 | E40 | O33-wzx | KJ755564 | O33:K-:H- | O33 | + |
| O034 | H304 | O34-wzx | KJ778803 | O34:K-:H10 | O34 | + |
| O035 | E77a | O35-wzx | FJ940774 | O35:K-:H10 | O35 | + |
| O036 | H502a | O36-wzx | AB811613 | O36:K-:H9 | O36 | + |
| O037 | 510c | O37-wzx | KJ755554 | O37:K-:H10 | O37 | + |
| O038 | F11621-41 | O38-wzx | KP710589 | O38:K-:H26 | O38 | + |
| O039 | H7 | O39-wzx | AB811616 | O39:K-:H- | O39 | + |
| O040 | H316 | O40-wzx | EU296417 | O40:K-:H4 | O40 | + |
| O041 | H710c | O42-wzx | AB811617 | O41:K-:H40 | O41 | + |
| O042 | P11a | O28ac-wzy | FJ539194 | O42:K-:H37 | O28ac, O42 | + |
| O043 | Bi7455-41 | O43-wzy | KJ778789 | O43:K-:H2 | O43 | + |
| O044 | H702c | O106-wzx | AB811620 | O44:K-:H18 | O106, O17, O44, O73, O77 | + |
| O045 | H61 | O45-wzx | AY771223 | O45:K1:H10 | O45 | + |
| O046 | P1c | O136-wzx | AB811621 | O46:K-:H16 | O134, O46 | + |
| O048 | U8-41 | O48-wzx | KJ710508 | O48:K-:H- | O48 | + |
| O049 | U12-41 | O49-wzx | AB811623 | O49:K+:H12 | O49 | + |
| O050 | U18-41 | O2-wzy | AB811624 | O50:K-:H4 | O2, O50 | + |
| O051 | U19-41 | O51-wzx | AB812020 | O51:K-:H24 | O51 | + |
| O052 | U20-41 | O52-wzt | AY528413 | O52:K-:H10 | O52 | + |
| O053 | Bi7327-41 | O53-wzx | EU289392 | O53:K-:H3 | O53 | + |
| O054 | Su3972-41 | O54-wzx | AB812085 | O54:K-:H2 | O54 | + |
| O056 | Su3684-41 | O56-wzx | DQ220293 | O56:K+:H- | O56 | + |
| O058 | F8962-41 | O58-wzy | EU294175 | O58:K-:H27 | O58 | + |
| O059 | F9095-41 | O59-wzy | AY654590 | O59:K-:H19 | O59 | + |
| O060 | Fl0167a-41 | O60-wzt | AB812022 | O60:K-:H33 | O60 | + |
| O061 | Fl0167b-41 | O61-wzx | GU220362 | O61:K-:H19 | O61 | + |
| O063 | Fl0598-41 | O63-wzy | EU549862 | O63:K-:H- | O63 | + |
| O064 | K6b | O64-wzx | AB812025 | O64:K-:H- | O64 | + |
| O065 | Klla | O65-wzy | KP710592 | O65:K-:H- | O65 | + |
| O066 | Pla | O66-wzx | DQ069297 | O66:K-:H25 | O66 | + |
| O068 | P7d | O62-wzx | KJ534585 | O68:K-:H4 | O62, O68 | + |
| O069 | P9b | O69-wzx | KJ778804 | O69:K-:H38 | O69 | + |
| O070 | P9c | O70-wzx | LC550088 | O70:K-:H42 | O70 | + |
| O071 | P10a | O71-wzy | GU445927 | O71:K-:H12 | O71 | + |
| O074 | E3a | O74-wzy | KJ778807 | O74:K-:H39 | O74 | + |
| O076 | E5d | O76-wzx | AB812031 | O76:K-:H8 | O76 | + |
| O077 | E10 | O106-wzx | DQ000314 | O77:K96:H- | O106, O17, O44, O73, O77 | + |
| O078 | E38 | O78-wzy | KJ778787 | O78:K?:H- | O78 | + |
| O080 | E71 | O80-wzy | AB812032 | O80:K-:H26 | O80 | + |
| O081 | H5 | O81-wzx | KJ778811 | O81:K97:H- | O81 | + |
| O082 | H14 | O82-wzy | AB812034 | O82:K-:H- | O82 | + |
| O083 | H17a | O83-wzy | KJ778808 | O83:K-:H31 | O83 | + |
| O084 | H19 (Knipschildt) | O84-wzy | KJ778809 | O84:K-:H21 | O84 | + |
| O085 | H23 | O85-wzy | KJ778791 | O85:K-:H | O85 | + |
| O086 | H35 | O86-wzy | AY670704 | O86:K-:H- | O86 | + |
| O089 | H68 | O89-glycosyl transferase family 2 protein | KJ755555 | O89:K-:H16 | O89 | + |
| O090 | H77 | O127-wzx | AY493508 | O90:K-:H- | O127, O90 | + |
| O091 | H307b | O91-wzy | AY035396 | O91:K-:H- | O91 | + |
| O092 | SSI 82099 | O92-wzt | AB812040 | O92:K-:H33 | O92 | + |
| O093 | 2885-1 | O93-wzy | AB812041 | O93 | O93 | + |
| O095 | H311a | O95-wzm | KJ755556 | O95:K+:H33 | O95 | + |
| O096 | H319 | O96-wzy | KJ778788 | O96:K-:H19 | O96 | + |
| O097 | H320a | O97-wzm | KJ778810 | O97:K-:H- | O97 | + |
| O098 | H501d | O98-wzx | DQ180602 | O98:K-:H8 | O98 | + |
| O099 | H504c | O99-wzm | FJ940773 | O99:K-:H33 | O99 | + |
| O100 | H509a | O100-wzx | KJ778805 | O100:K-:H33 | O100 | + |
| O101 | H510a | O101-putative S-adenosylmethionine-dependent methyltransferase | KJ778806 | O101:K-:H33 | O101, O162 | + |
| O102 | H511 | O102-wzy | JX087966 | O102:K-:H40 | O102 | + |
| O103 | H515b | No hits | AY532664 | O103:K+:H8 | O103 | + |
| O104 | H519 | No hits | AF361371 | O104:K-:H12 | O104 | + |
| O105 | H520b | O105-wzx | EU294171 | O105:K-:H8 | O105 | + |
| O106 | H521a | O106-wzx | DQ000315 | O106:K-:H33 | O106, O17, O44, O73, O77 | + |
| O107 | H705 | O107-wzx | EU694095 | O107:K98:H27 | O107, O117 | + |
| O108 | H708b | O108-wzx | KP710597 | O108:K-:H10 | O108 | + |
| O109 | H709c | O109-wzy | HM485572 | O109:K-:H19 | O109 | + |
| O111 | Stoke W | O111-wzx | AF078736 | O111:K?:H- | O111 | + |
| O112ab | 1411-50 | O112ac-wzx | EU296413 | O112ab:K?:H18 | O112ab | + |
| O113 | 6182-50 (=32w) | O113-wzy | AF172324 | O113:K?:H21 | O113 | + |
| O114 | KlO (=26w=H34w) | O114-wzx | AY573377 | O114:K?:H32 | O114 | + |
| O115 | 27w | O115-wzx | GU068041 | O115:K-:H18 | O115 | + |
| O116 | 28w | O116-wzx | AB812051 | O116:K+:H10 | O116 | + |
| O117 | 30w | O107-wzx | EU694096 | O117:K98:H4 | O107, O117 | + |
| O119 | 34w | O119-wzx | GQ499368 | O119:K?:H27 | O119 | + |
| O120 | 35w | O120-wzx | AB812052 | O120:K18a:H6 | O120 | + |
| O121 | 39w | O121-wzx | AY208937 | O121:K-:H12 | O121 | + |
| O123 | 43w | O123-wzx | DQ676934 | O123:K-:H16 | O123, O186 | + |
| O124 | 227 | O164-wzx | EU296420 | O124:K?:H30 | O124, O164 | + |
| O125ac | 2129-54 | O125ab-wzx | KP835695 | O125ac:K-:H12 | O125ab, O125ac | + |
| O127 | 4932-53 | O127-wzx | AY493508 | O127:K?:H- | O127, O90 | + |
| O128 | 56-54 | OX38-wzx | AY217096 | O128ab:K?:H2 | O128, OX38 | + |
| O129 | 178-54 | O13-wzy | EU296424 | O129:K-:H11 | O129, O13, O135 | + |
| O130 | 4866-53 | O130-wzy | EU296421 | O130:K-:H9 | O130 | + |
| O131 | S239 (=H27w) | O131-wzx | KJ755544 | O131:K+:H28 | O131 | + |
| O132 | N87 (=H30w) | O132-wzx | KJ755553 | O132:K+:H28 | O132 | + |
| O133 | N282 (=H31w) | O133-wzx | KJ710509 | O133:K-:H29 | O133 | + |
| O134 | 4370-53 | O134-wzx | KJ755545 | O134:K-:H35 | O134, O46 | + |
| O135 | Coli Pecs | O13-wzy | EU296423 | O135:K-:H- | O129, O13, O135 | + |
| O136 | 1111-55 | O136-wzy | KJ755546 | O136:K?:H- | O136 | + |
| O137 | RVC1787 | O137-wzx | KJ755547 | O137:K?:H41 | O137 | + |
| O138 | CDC62-57 | O138-wzx | DQ109551 | O138:K?:H14 | O138 | + |
| O139 | CDC63-57 | O139-wzy | KJ755548 | O139:K12:H1 | O139 | + |
| O140 | 149-51 | O140-wzy | KJ755552 | O140:K-:H43 | O140 | + |
| O141 | E68 | O141-wzx | DQ868765 | O141:K-:H4 | O141 | + |
| O142 | C771 | O142-wzx | KJ755549 | O142:K?:H6 | O142 | + |
| O144 | 1624-56 | O144-wzx | KJ755550 | O144:K-:H- | O144 | + |
| O145 | E1385(3) | O145-wzx | AY647260 | O145:K-:H- | O145 | + |
| O146 | CDC2950-54 | O146-wzx | DQ465249 | O146:K-:H21 | O146 | + |
| O147 | G1253 | O147-wzx | DQ868766 | O147:K?:H19 | O147 | + |
| O148 | E519-66 | O148-wzy | DQ167407 | O148:K-:H28 | O148 | + |
| O149 | Al | O149-putative polysaccharide pyruvyl transferase | DQ091854 | O149:K?:H10 | O149 | + |
| O150 | 1935 | O150-wzx | EU294168 | O150:K93:H6 | O150 | + |
| O151 | 880-67 | O118-wzx | DQ990685 | O151:K-:H10 | O118, O151 | + |
| O152 | 1184-68 | O152-wzx | EU294170 | O152:K-:H- | O152 | + |
| O153 | 14097 | O153-wzy | KJ755551 | O153:K-:H7 | O153 | + |
| O154 | E1541-68 | O154-wzx | AB812064 | O154:K94:H4 | O154 | + |
| O155 | E1529-68 | O155-wzy | AB812064 | O155:K-:H9 | O155 | + |
| O156 | E1585-68 | O156-wzx | AB812064 | O156:K-:H47 | O156 | + |
| O157 | 4288-84 | O157-wzx | AF061251 | O157:K?:H7 | O157 | + |
| O158 | E1020-72 | O158-wzx | GU068044 | O158:K-:H23 | O158 | + |
| O159 | E2476-72 | O159-wzy | EU294176 | O159:K-:H20 | O159 | + |
| O161 | E223-69 | O161-wzy | GU220361 | O161:K-:H54 | O161 | + |
| O163 | SN38-1 | OX21-wzy | KP710593 | O163:K-:H19 | O163, OX21 | + |
| O164 | DRL145-46 | O164-wzx | EU296420 | O164:K-:H- | O124, O164 | + |
| O165 | E78634 | O165-wzx | GU068045 | O165:K-:H- | O165 | + |
| O166 | 3866-54 | O166-wzx | GU299794 | O166:K-:H4 | O166 | + |
| O167 | E10702 | O167-wzx | EU296408 | O167:K-:H5 | O167 | + |
| O168 | E10710 | OX6-wzy | EU296403 | O168:K-:H16 | O168, OX6 | + |
| O169 | 1792-54 | O169-wzx | KJ778796 | O169:K-:H8 | O169 | + |
| O170 | 745-54 | O170-wzy | KJ778797 | O170:K-:H1 | O170 | + |
| O171 | 198 | O171-wzy | KJ739598 | O171:K-:H2 | O171 | + |
| O172 | 3288-85 | O172-wzy | AY545992 | O172:K-:H- | O172 | + |
| O173 | L119B-10 | O173-wzy | GU068046 | O173:K-:H- | O173 | + |
| O174 | 2531-54 | O174-wzy | DQ008592 | O174:K-:H27 | O174 | + |
| O175 | 2533-54 | O175-wzy | KJ739597 | O175:K-:H28 | O175 | + |
| O176 | E29518-83 | O176-wzy | KJ778798 | O176:K?:H- | O176 | + |
| O178 | E54071-88 | O178-haloacid dehalogenase-like hydrolase | KJ778799 | O178:K?:H7 | O178 | + |
| O179 | E43478 | O179-wzy | KJ778800 | O179:K?:H8 | O179 | + |
| O180 | 86-381 | O180-wzx | JQ751058 | O180:K?:H- | O180 | + |
| O181 | 92-1250 | O181-wzx | KJ778801 | O181:K?:H49 | O181 | + |
| O182 | SSI 81930 | O182-wzx | KJ778802 | O182:K48:H25 | O182 | + |
| O183 | 99-2442 | O183-wzx | AB627352 | O183:H18 | O183 | + |
| O184 | 99-4473 | OX9-wzx | KP710595 | O184:K-:H11 | O184, OX9 | + |
| O185 | 99-6301 | O185-wzy | AB812081 | O185:H28 | O185 | + |
| O187 | SSI 81829 | O187-wzx | KJ739600 | O187:K-:H52 | O187 | + |
| OX13 | 22-56 | OX13-wzx | KP710591 | OX13:K?:H? | OX13 | + |
| OX19 | PDL-39A | O11-wzx | KP868751 | OX19:K?:H21 | OX19, O11 | + |
| OX21 | Pur 599 | OX21-wzy | KJ739596 | OX21:K?:H14 | O163, OX21 | + |
| OX25 | F6432 | OX25-wzy | KP710594 | OX25:H6 | OX25 | + |
| OX28 | 7026N | OX28-glycosyl transferase family 2 | KT207929 | OX28:K?:HNM | OX28 | + |
| OX38 | PF11-6E | OX38-wzx | KJ739599 | OX38:K?:H47 | O128, OX38 | - |
| OX43 | 8547 | O19-wzx | KP835691 | OX43:K?:H19 | O19, OX43 | + |
| OX9 | 3461-54 | OX9-wzx | KJ778795 | OX9:K?:H? | O184, OX9 | + |

**Supplementary Table S2.** AgriSeq H-typing results of H-type standard strains.

| Strain no. | Strain Designation | Expressed flagellin gene | Additional flagellin gene | Accession no. | Serotype | AgriSeq call *^a^* | *E. coli* spp. *^b^* |
| --- | --- | --- | --- | --- | --- | --- | --- |
| H01 | Su1242 | *fliC*_H1_ | - | AB028471 | O2:K2ab:H1 | H1 | + |
| H02 | Bi7455-41 | *fliC*_H2_ | - | AY249138 | O43:K-:H2 | H2 | + |
| H03 | Bi7327-41 | *flkA*_H3_ | *fliC*_H16_ | AB128916 | O53:K-:H3 | H16, H3 | + |
| H05 | U4-41 | *fliC*_H5_ | - | AB028473 | O4:K3:H5 | H5 | + |
| H06 | A 20a | *fliC*_H6_ | - | AY249991 | O2:K1:H6 | H6 | + |
| H07 | U5-41 | *fliC*_H7_ | - | AB028474 | O1:K1:H7 | H7 | + |
| H08 | App.320c | *fliC*_H8_ | - | AJ567918 | O2:K-:H8 | H8 | + |
| H09 | Bi7575-41 | *fliC*_H9_ | - | AY249994 | O8:K25:H9 | H9 | + |
| H10 | Bi623-42 | *fliC*_H10_ | - | AY249995 | O11:K10:H10 | H10 | + |
| H11 | Su4321-41 | *fliC*_H11_ | - | AY249996 | O13:K11:H11 | H11 | + |
| H12 | Bi316-42 | *fliC*_H12_ | - | AY249997 | O9:K9:H12 | H12 | + |
| H14 | F10018-41 | *fliC*_H14_ | - | AY249998 | O18ab:K-:H14 | H14 | + |
| H15 | E39a | *fliC*_H15_ | - | AY249999 | O23:K18ab:H15 | H15 | + |
| H16 | F8316-41 | *fliC*_H16_ | - | AY250000 | O6:K15:H16 | H16 | + |
| H19 | A18d | *fliC*_H19_ | - | AY250002 | O9:K36:H19 | H19 | + |
| H20 | H330b | *fliC*_H20_ | - | AY250003 | O8:K49:H20 | H20 | + |
| H21 | SSI 81942 | *fliC*_H21_ | - | AY337484 | O8:K49:H21 | H21 | + |
| H24 | K72 (H25w) | *fliC*_H24_ | - | AY250006 | O51:K12:H24 | H24 | + |
| H26 | S239 (=H27w) | *fliC*_H26_ | - | AY250008 | O131:K-:H26 | H26 | + |
| H27 | K50 (=H28w) | *fliC*_H27_ | - | AY250009 | O15:Kne:H27 | H27 | + |
| H28 | N87 (=H30w) | *fliC*_H28_ | - | AY250010 | O132:K+:H28 | H28_2, H28_1 | + |
| H29 | N282 (=H31w) | *fliC*_H29_ | - | AY250012 | O133:K-:H29 | H29 | + |
| H30 | N157 (=H32w) | *fliC*_H30_ | - | AY250011 | O38:Kne:H30 | H30 | + |
| H31 | K15 (=H33w) | *fliC*_H31_ | - | AY250013 | O3:K-:H31 | H31 | + |
| H32 | SSI 85351 | *fliC*_H32_ | - | AY250014 | O114:H32 | H32 | + |
| H34 | SSI 81950 | *fliC*_H34_ | - | AY250016 | O86:H34 | H34 | + |
| H37 | P11a | *fliC*_H37_ | - | AY250017 | O42:K-:H37 | H37 | + |
| H38 | P9b | *fliC*_H38_ | - | AY250018 | O69:K-:H38 | H38 | + |
| H39 | E3a | *fliC*_H39_ | - | AY250019 | O74:K-:H39 | H39 | + |
| H40 | E49 | *fliC*_H40_ | - | AJ884568 | O79:K-:H40 | H40 | + |
| H41 | RVC1787 | *fliC*_H41_ | - | AY250020 | O137:H41 | H41 | + |
| H42 | P9c | *fliC*_H42_ | - | AY250021 | O70:K-:H42 | H42 | + |
| H43 | 149-51 | *fliC*_H43_ | - | AY250022 | O140:K-:H43 | H43 | + |
| H45 | 4106-54 | *fliC*_H45_ | - | AY250023 | O52:Kne:H45 | H45 | + |
| H46 | 5306-56 | *fliC*_H46_ | - | AY250024 | O26:H46 | H46 | + |
| H47 | 1755-58 | *flkA*_H47_ | *fliC*_H21_ | EF392694 | O86:Kne:H47 | H21, H47 | + |
| H49 | 2147-59 | *fliC*_H49_ | - | AY250026 | O6:K13:H49 | H49 | + |
| H51 | SSI 81958 | *fliC*_H51_ | - | AB028481 | O8:K50:H51 | H51 | + |
| H54 | SSI 85360 | *flmA*_H54_ | *fliC*_H21_ | AB128918 | O161:K-:H54 | H21, H54 | + |
| H55 | E2987-73 | *fllA*_H55_ | *fliC*_H38_ | EF165537 | O75:Kne:H55 | H55, H38 | + |
| H56 | SN2N-1 | *fliC*_H56_ | *-* | AY250029 | O139:K-:H56 | H56 | + |
| *^a^* Both H28 primers amplify targets in the H28 standard strain *fliC* gene.  *^b^* The H56 standard strain was negative for the *E. coli* species target in a single repetition of the AgriSeq assay. | | | | | | | |

**Supplementary Table S3. Reference sequences used for the design of STEC virulence gene primers.**

| STEC virulence gene | Reference sequences used for primer design |
| --- | --- |
| *stx*_1_ | KP203839.1, KP120708.1, KP120709.1, KP120710.1, KP120711.1, KP120712.1, KP120713.1, KP120714.1, KP120715.1, KP120716.1, JX206444.1, JQ327853.1, JQ327854.1, GQ429154.1, GQ429155.1, GQ429156.1, GQ429157.1, GQ429158.1, GQ429159.1, GQ429160.1, EU273279.1, AM230662.1, AM230663.1, AY986980.1, AY986981.1, AY986982.1, AB050958.1, AB050959.1, AB083043.1, AB083044.1, AY170851.1, AY135685.1, AF461166.1, AF461168.1, AF461169.1, AF461172.1, AJ312232.1, AJ314838.1, AJ314839.1, AB030485.1, AB012101.1, AB012102.1 |
| *stx*_2_ | KP120717.1, KP120718.1, KP120719.1, KP120720.1, KP120721.1, KP120722.1, KP120723.1, KP120724.1, KP120725.1, KP120726.1, KM516094.1, KM516095.1, KM516096.1, KM516097.1, KM516098.1, KM516099.1, KJ158456.1, AB499801.1, AB499802.1, AB499803.1, AB499807.1, AB499808.1, AB499809.1, AB499810.1, AB499811.1, AB499812.1, AB499813.1, AB499814.1, HF558442.1, HF558443.1, HF558444.1, HF558445.1, KF932358.1, KF932359.1, KF932360.1, KF932361.1, KF932362.1, KF932363.1, KF932364.1, KF932365.1, KF932366.1, KF932367.1, KF932368.1, KF932369.1, KF932370.1, KF932371.1, KF932372.1, KF932373.1, KF932374.1, KF932375.1, KF932376.1, KF932377.1, KF932378.1, AB854278.1, AB854279.1, AB854280.1, AB854281.1, AB854282.1, AB854283.1, AB854284.1, AB854285.1, AB854286.1, AB854287.1, AB854288.1, AB854289.1, AB854290.1, KC659956.1, KC339670.2, JX206445.1, JQ411011.1, FR850031.1, FR850032.1, FR850033.1, FR850034.1, GU126552.1, FR874039.1, FR874040.1, FR874041.1, FR851896.1, FR851897.1, FR851898.1, FN252457.1, FN252458.1, FN252459.1, GU228505.1, GQ995452.1, GQ919289.1, GU983682.2, GU983683.2, FM998838.1, FM998839.1, FM998840.1, FM998841.1, FM998842.1, FM998843.1, FM998844.1, FM998845.1, FM998846.1, FM998847.1, FM998848.1, FM998849.1, FM998850.1, FM998851.1, FM998852.1, FM998853.1, FM998854.1, FM998855.1, FM998856.1, FM998857.1, FM998858.1, FM998859.1, FM998860.1, FM998861.1, FN182284.1, FN182285.1, FN182286.1, FN182287.1, GQ429161.1, GQ429162.1, GQ429163.1, GQ429164.1, GQ429165.1, GQ429166.1, GQ429167.1, GQ429168.1, GQ429169.1, GQ429170.1, GQ429171.1, GQ429172.1, FM177471.1, FM177472.1, AM982821.2, EU999145.1, EU999146.1, EU999147.1, EU999148.1, EU999149.1, EU999150.1, EU999151.1, EU999152.1, EU999153.1, EU999154.1, AB290936.1, AB290937.1, AB290938.1, AM904726.1, EU086525.1, EF584538.1, AM230664.1, AB252836.1, AB168103.1, AB168104.1, AB168105.1, AB168106.1, AB168107.1, AB168108.1, AB168109.1, AB168110.1, AB168111.1, AB232172.1, DQ143180.1, DQ143181.1, DQ143182.1, DQ143183.1, AJ966782.1, AJ966783.1, AY652745.1, AY368993.1, AY095209.1, AF500187.1, AF500188.1, AF500189.1, AF500190.1, AF500191.1, AF500192.1, AF500193.1, AF479828.1, AF479829.2, AF461165.1, AF461167.1, AF461170.1, AF461171.1, AF461173.1, AF461174.1, AB071845.1, AJ313016.1, AF329817.1, AB046175.1, AB030484.1, AJ251483.1, AJ270998.1, AF291819.1, AJ010730.1, AF162758.1, AB017524.1 |
| *eae* | AB647618.1, FJ609798.1, FJ609799.1, FJ609800.1, FJ609801.1, FJ609802.1, FJ609803.1, FJ609804.1, FJ609805.1, FJ609806.1, FJ609807.1, FJ609808.1, FJ609809.1, FJ609810.1, FJ609811.1, FJ609812.1, FJ609813.1, FJ609814.1, FJ609815.1, FJ609816.1, FJ609818.1, FJ609820.1, FJ609822.1, FJ609823.1, FJ609828.1, FJ609829.1, FM872416.1, FM872417.1, FM872418.1, FM872419.1, FM872420.1, FM872421.1, FM872422.1, FM872423.1, FM872424.1, FM872425.1, FM872426.1, AB334558.1, AB334559.1, AB334560.1, AB334561.1, AB334562.1, AB334563.1, AB334564.1, AB334565.1, AB334566.1, AB334567.1, AJ877226.1, AJ877227.1, AJ877228.1, AJ877229.1, AJ877230.1, AM116755.1, EF204930.1, AJ833637.1, AJ833638.1, DQ523600.1, DQ523601.1, DQ523602.1, DQ523603.1, DQ523604.1, DQ523605.1, DQ523606.1, DQ523607.1, DQ523609.1, DQ523610.1, DQ523611.1, DQ523612.1, DQ523613.1, DQ523614.1, AM180621.1, AJ879898.1, AJ879899.1, AJ879900.1, AJ876647.1, AJ876648.1, AJ876649.1, AJ876650.1, AJ876651.1, AJ876652.1, AJ876653.1, AJ876654.1, AJ875027.1, AJ781125.1, AJ748082.1, AJ748083.1, AJ748084.1, AJ744865.1, AJ715407.1, AJ715408.1, AJ715409.1, AJ705049.1, AJ705050.1, AJ705051.1, AJ705052.1, AY255520.1, AY223510.1, AJ308550.1, AF449414.1, AF449415.1, AF449419.1, AF449420.1, AJ275089.1, AJ275090.1, AJ275091.1, AJ275092.1, AJ275093.1, AJ275094.1, AJ275095.1, AJ275096.1, AJ275097.1, AJ275098.1, AJ275099.1, AJ275100.1, AJ275101.1, AJ275102.1, AJ275103.1, AJ275104.1, AJ275105.1, AJ275106.1, AJ275107.1, AJ275108.1, AJ275109.1, AJ275110.1, AJ275111.1, AJ275112.1, AJ275113.1, AF253560.1, AF253561.1, AJ271407.1, AJ298279.1, AB040740.1, AF065628.1, AF081182.1, AF081183.1, AF081184.1, AF081185.1, AF081186.1, AF081187.1, AF022236.1, U66102.1, L06255.1, M58154.1, Z11541.1 |
